# A Case of Acute Albuminuria

**Published:** 1886-03

**Authors:** C. H. H. Hall

**Affiliations:** Surgeon U. S. Navy; Washington, D. C.


### A CASE OF ACUTE ALBUMINURIA. *By* C. H. H. HALL, P. A., *Surgeon U. S. Navy.*

[Read before the Naval Medical Society.]

The following case occurred on board the U. S. S. Ranger, while *en route* from the west coast of Mexico to San Francisco. As it is read solely to introduce an exchange of experience upon an important point in therapeutics, viz., the use of opium in albuminuria, the paper is merely an outline history of the case.

J. M., second-class fireman, age 41, native of Java, while returning from liberty on shore, and partially intoxicated, fell from the wharf, at San Diego, California, into the bay. He remained clinging to a pile, and exposed to a chill wind, for nearly an hour before his rescue by a boat from the ship. When brought on board he was considerably fatigued and thoroughly chilled, but sober. His clothing was changed at once, and he was put into a hammock.

Next morning he appeared at sick-call, complaining of pain in the left hip, which had been bruised in falling. He had also a feverish air, and slight rise of temperature, 100.2° F. under the tongue.

Injected morphia grain ¼ near the bruise, and directed patient to remain in his hammock. At 5 P. M. temperature was 99.2° F. On the 2nd day the morning temperature was 101.6° F.; at 5 P.M. 104.2° F. He had a marked chill at 2 P. M., without complaint of local distress. Examination of the chest discovered nothing wrong. As he had recently recovered from malarial fever, the exposure was suspected to have induced a recurrence of it. He received quinine.

On the 3rd day he had one thin stool during the night; the morning temperature was 104.4° F.; he had nausea, thirst and dry, hot skin; there was no abdominal tenderness. At midday the temperature was 104.6° F., and the patient was vomiting. The urine was slightly albuminous; specific gravity was 1.019, acid, dark-colored. Fluid extract of jaborandi ℥i. caused profuse sweating, with fall of temperature to 102.2° F. at 3 P.M., and 102° F. at 5 P.M. By 8 P.M., however, the temperature had risen to 103.4° F., and the patient was vomiting again. These symptoms of suspected remittent fever were not accompanied by the usually concomitant browache. But neither this fact nor the presence of albumen in the urine, which had been lately observed in otherwise ordinary cases, was remarked as significant of error in diagnosis. The vomiting was checked by hypodermic injection of morphine over the stomach, and later quinine was resumed. On the 4th day the morning temperature was 102.6° F.; at 2 P.M., 101.8° F.; the urine passed at this time was dark colored, turbid, of specific gravity of 1.025, and slightly albuminous. An inferior microscope revealed no casts. Quinine was continued to considerable cinchonism, and slight nausea by evening-time. The urine having become scanty, a diuretic mixture of digitalis, acetate of ammonium and acetate of potash was ordered. Later, when gastric irritability threatened, a suspension of stomach medication was ordered, and morphia gr. 1/4 was injected, hypodermically, with satisfactory result. The bowels were moved normally. On the 5th day the morning temperature was 103.2° F., and the evening, 103° F. Late in the afternoon the patient complained of a "stitch" near the right nipple. Auscultation revealed over the right mammary region a rubbing sound with respiratory movements. There were no other chest signs, and the respirations were tranquil. Injected morphine, gr. 1/8, near the nipple, after which there was no recurrence of the pain. He had 36 grains of quinine during the day, and the diuretic mixture was continued. On the 6th day the morning temperature was 102.2° F. There was no stool for two days. A simple enema was, shortly after its administration, voided without the patient's consciousness. One hour later he passed, normally,.a thin, light-yellow, stool. The urine at the same was rather turbid, dark-colored, acid; specific gravity 1.020, and slightly albuminous. Muttering delirium came on during the middle of the day, and the temperature rose to 104° F. Jaborandi and wet-pack brought it down to 102.4° F., and put the patient to tranquil sleep. Within eight hours after using the wet-pack he passed two thin stools and a large quantity of urine. The evening temperature was 104° F. Quinine. On the 7th day the morning temperature was 102.8° F. The patient was restless and irritable. About 1 P.M., the temperature being 101.4° F., he suddenly became maniacal; requiring considerable force to keep him in the cot. Injected hypodermically grain $\frac{1}{3}$ of morphine. He became tranquil and rational at once, and a half hour later, after saying he felt much better, he fell into quiet sleep. As nothing in his condition indicated other than easy slumber, he was not disturbed until 6 P.M., but he was then found to be comatose, could not be aroused, and the reflex sensibility of the conjunctivæ was slight. Note the following: pulse 90, full and rather strong, not irregular; respirations 23½, of fair character, except slightly hissing stertor; temperature in axilla 100.4° F. (buccal temperature at same time yesterday was 104.° F.); skin warm, dry, and, if at all changed, rather pale; pupils small, immobile, and unequal, the left being the larger; conjunctivæ scarcely sensitive. A catheter, introduced to determine whether the kidneys were acting, brought away but a few drops of turbid, highly albuminous urine. Counter-irritation failing to arouse him by 7:30 P.M., atropine, grain $\frac{1}{20}$, was injected, hypodermically. At this time the axillary temperature was 100.6° F., respiration 23½, pulse 90. Liquids put into the open mouth were swallowed tardily, and by reflex action only; the bowels were evacuated into the bed, the conjunctivæ were quite insensitive, tracheal *râles* were present, the extremities were cool, and there was general muscular relaxation. As the pupils showed no effect of atropine at 8 P. M., *i. e.*, one half-hour after its administration, the drug was repeated in half the quantity, grain $\frac{1}{40}$. The pupils began very soon to dilate.

At 9 P. M. the respirations were 24, full and vigorous, pulse 120, of good strength but less volume, axillary temperature 101.8° F., and pupils much dilated. A catheter brought away one ounce of turbid urine with one-fourth albumen. Jaborandi, put into the mouth, was tardily swallowed with a start and a moan. Auscultation of the chest discovered dry rubbing sounds in the left lower axillary region. Applied dry cups over the kidneys. 9:30 P. M., axillary temperature was 102.6° F., respiration 24, extremities warm, light brow-sweat from the jaborandi, ℥ss. Gave him the diuretic mixture previously mentioned. 11:30 P. M., axillary temperature 103.8° F., respiration 31, pulse 120, skin hot and dry. Patient opened his eyes when his name was called, and swallowed the diuretic, though with some difficulty, which was attributed to the action of the atropine upon the pharynx. At midnight drew off by catheter fifteen ounces of urine, turbid, acid, specific gravity 1.020, moderately (about one-sixth) albuminous. The patient now steadily improved to complete consciousness at 4 A. M., at which time the respirations were 36, pulse 120, axillary temperature 103.4° F., skin warm but dry, and kidneys active, fourteen ounces more of urine having been drawn off at 2 A. M. The diuretic was continued each two hours. 8th to 10th days. Patient visibly improved, though the temperature continued but little abated (101°–103° F.). The albumen in the urine diminished to a haze, the kidneys were active, the bowels easy, and gastric irritability absent. The pulse remained rapid, 102–111, and respirations frequent but easy. The microscope discovered in the urine, immediately after its evacuation, many minute oil globules and a large number of pellucid globular bodies, whose nature was not determined. The diuretic was continued on the 11th day. The morning temperature in mouth was 100° F., pulse 92. The patient was cheerful, took food with relish, and had no disturbing symptom. The urine was passed in good quantity, hazy only with albumen, clear, acid, specific gravity 1.019, and the patient was apparently on the verge of convalescence. But on account of unavoidable exposure to varying temperature in the sick-bay, it was thought best to transfer him to a hospital. Accordingly he was warmly wrapped in blankets, placed in a tented cot, which was enclosed on all sides from possible drafts, transferred to a closed ambulance wagon on the navy yard dock and sent to the naval hospital, Mare Island, about one mile distant. These precautions against chill were taken, notwithstanding his reiterated request to be allowed to walk to the wagon and thus show how much better he was. Three days after his admission to hospital he died in high fever. No necropsy.

*Remarks.*—Three points in this case deserve special notice:

1. The differential diagnosis of the coma.

2. The action of the drugs, jaborandi and atropine.

3. The agency of the morphine in the occurrence of the supression of urine.

1. Was the coma due to narcosis, to uræmia, or to both? These were the symptoms: slight hissing stertor, respirations 23½, pulse .90 and substantially normal, temperature (axilla) 100.4° F., one degree less than immediately before and after the attack, face rather pale than flushed. These are not characteristic of opium narcosis. To them add the actual presence of uræmic delirium before the drug was given. As to the state of the pupils, moderately contracted and unequal, it is enough to say that authorities differ upon the state of the pupils in uræmia, when the symptom is mentioned at all. I do not think the morphine had more than a secondary agency in the causation of the coma.

2. Of the drugs, I wish to note merely the considerable fall of temperature that followed the administration of the jaborandi, and the speedy rise, the short duration of this effect being an objection to the use of the drug as a febrifuge.

I have seen but little mention of the use of atropine in connection with the therapeutics of anuria, although Harley wrote, in 1869, ("The Old Vegetable Neurotics," p. 213), "Belladonna indeed is, in the truest sense of the word, a diuretic, and more powerful perhaps than any other that we possess." Its action upon the bladder, obtunding its sensibility, may be an objection to its common use as a diuretic, but this objection loses force in the presence of suppression of urine, and of a physician with a catheter to anticipate retention. In the case related above, I think atropine was the agent of relief, and not by antagonizing the effect upon the central nervous system of a third of a grain of morphine, but by its specific action upon the kidneys.

3. It is not long ago since a question about the use of opium in albuminuria would have received but one answer, enjoining the greatest caution against the hazard of it. In 1875, however, Professor Loomis, of New York ("Lectures on Diseases of the Respiratory Organs, Heart and Kidneys," p. 450), challenged the believers in this therapeutic maxim, saying, "In 1868," etc., and in his "Text-book of Practical Medicine," lately published, this opinion in favor of the use of opium in uræmia is declared with emphasis. This declaration has led the profession to re-examine the question, and results of this study sustain Professor Loomis' opinion.

WASHINGTON, D. C., 11 February, 1886.

